# Effect of stigma reduction intervention strategies on HIV test uptake in low- and middle-income countries: a realist review protocol

**DOI:** 10.1186/s13643-015-0130-3

**Published:** 2015-11-02

**Authors:** Subash Thapa, Karin Hannes, Margaret Cargo, Anne Buve, Catharina Mathei

**Affiliations:** Department of Public Health and Primary Care, KU Leuven, Kapucijnenvoer 33, 3000 Leuven, Belgium; Department of Public Health, Institute of Tropical Medicine, Nationalestraat 155, 2000 Antwerp, Belgium; Centre for Sociology Research, Faculty of Social Sciences, KU Leuven, 3000 Leuven, Belgium; School of Population Health, University of South Australia, Adelaide, SA 5001 Australia

**Keywords:** Realist synthesis, HIV stigma, Stigma reduction intervention strategies, HIV test uptake, Low- and middle-income countries

## Abstract

**Background:**

Several stigma reduction intervention strategies have been developed and tested for effectiveness in terms of increasing human immunodeficiency virus (HIV) test uptake. These strategies have been more effective in some contexts and less effective in others. Individual factors, such as lack of knowledge and fear of disclosure, and social-contextual factors, such as poverty and illiteracy, might influence the effect of stigma reduction intervention strategies on HIV test uptake in low- and middle-income countries. So far, it is not clearly known how the stigma reduction intervention strategies interact with these contextual factors to increase HIV test uptake. Therefore, we will conduct a review that will synthesize existing studies on stigma reduction intervention strategies to increase HIV test uptake to better understand the mechanisms underlying this process in low- and middle-income countries.

**Methods:**

A realist review will be conducted to unpack context-mechanism-outcome configurations of the effect of stigma reduction intervention strategies on HIV test uptake. Based on a scoping review, we developed a preliminary theoretical framework outlining a potential mechanism of how the intervention strategies influence HIV test uptake. Our realist synthesis will be used to refine the preliminary theoretical framework to better reflect mechanisms that are supported by existing evidence. Journal articles and grey literature will be searched following a purposeful sampling strategy. Data will be extracted and tested against the preliminary theoretical framework. Data synthesis and analysis will be performed in five steps: organizing extracted data into evidence tables, theming, formulating chains of inference from the identified themes, linking the chains of inference and developing generative mechanisms, and refining the framework.

**Discussion:**

This will be the first realist review that offers both a quantitative and a qualitative exploration of the available evidence to develop and propose a theoretical framework that explains why and how HIV stigma reduction intervention strategies influence HIV test uptake in low- and middle-income countries. Our theoretical framework is meant to provide guidance to program managers on identifying the most effective stigma reduction intervention strategies to increase HIV test uptake. We also include advice on how to effectively implement these strategies to reduce the rate of HIV transmission.

**Systematic review registration:**

PROSPERO CRD42015023687

**Electronic supplementary material:**

The online version of this article (doi:10.1186/s13643-015-0130-3) contains supplementary material, which is available to authorized users.

## Background

Human immunodeficiency virus (HIV) stigma is a major barrier for HIV testing [1]. Testing for HIV can itself lead to stigma due to negative social perceptions about the test [2, 3]. Moreover, people fear that stigma of a positive HIV test result may lead to consequences, such as loss of friendship and family ties, dismissal from school and occupation, and denial from health care [2, 3]. Most importantly, the fear of getting tested for HIV might cause delayed access to treatment and care, which leads to higher transmission and lower survival rates [4, 5]. Therefore, it is very important that stigma reduction intervention strategies that are effective to increase HIV test uptake be identified and implemented.

Several intervention strategies to reduce HIV stigma have been developed and tested [6–9]. Moreover, studies [6, 10, 11] from high-income countries have reported that these intervention strategies have been effective not only to reduce stigma but also to increase HIV test uptake. However, findings from studies conducted in low- and middle-income countries indicate that such intervention strategies are effective to increase HIV disclosure and safer sex practices, but not to increase HIV test uptake [12–14]. Thus far, it is not known why stigma reduction intervention strategies are more effective in one context and less effective in another to increase HIV test uptake and also what exactly influences the fact that strategies are more or less effective [15].

Stigma reduction is very complex, and its impact on the behavior of people might be influenced by various individual and social-contextual factors [16–19]. Especially in low- and middle-income countries, individual factors, such as lack of knowledge, fear of HIV infection, fear of disclosure, and belonging to high-risk populations, influence the effect of stigma reduction on HIV test uptake. Likewise, social-contextual factors, such as poverty, illiteracy, lack of availability of treatment, and cultural and gender norms, impact on this process [16–19]. We expect the individual factors to be determined and controlled by social-contextual factors. If this is true, then the interaction between both would also influence the outcome of stigma reduction intervention strategies. This potential causal chain is yet to be explored in reviews focusing on low- and middle-income countries.

So far, realist synthesis that unpacks stigma reduction and HIV test uptake into context-mechanism-outcome (CMO) configurations has not been conducted. The mechanisms by which stigma reduction intervention strategies impact on HIV test uptake should be clarified, as this information is imperative to design appropriate strategies to increase HIV test uptake in low- and middle-income countries [20, 21]. Therefore, we will conduct a realist review that will synthesize existing studies to understand how one or more individual and social-contextual factors influence the effect of stigma reduction intervention strategies on HIV test uptake in low- and middle-income countries. Through this realist review, our aim is to develop and propose a theoretical framework that will guide program managers to identify the most effective stigma reduction intervention strategies to increase HIV test uptake and to effectively implement them to reduce the rate of HIV transmission.

### Aims of the review

Our aim is to develop and propose a theoretical framework that depicts CMO configurations of the effect of stigma reduction intervention strategies on HIV test uptake. Specifically, we ask the following realist synthesis question: How and under what circumstances do stigma reduction intervention strategies influence HIV test uptake? Our specific objectives are to

1. Document the different sorts of stigma reduction intervention strategies.
2. Identify effective intervention strategies to increase HIV test uptake.
3. Explain the mechanisms that generate the outcome of HIV test uptake.
4. Investigate the contextual variations in which the corresponding mechanisms and outcome are generated.
5. Develop a realist theoretical framework that uses CMO configuration to explain the effect of stigma intervention strategies on HIV test uptake.

## Methods

A realist perspective is chosen because it allows the evaluation of complex social interventions [22]. It is a theory-driven and multi-method-based research methodology that uses an interpretive approach to synthesize evidence to reveal how intervention strategies interact with contexts to trigger mechanisms and produce outcomes. At first, it aims to develop a program theory that explains how context influences mechanisms to generate outcomes. The preliminary theory is represented as a set of CMO configurations [22]. It then empirically tests this theory to investigate whether, why, or how intervention strategies produce observed outcomes, for whom and in what circumstances.

This realist review will integrate both quantitative and qualitative data to comprehensively understand how stigma reduction intervention strategies work, in what circumstances and for whom, to increase HIV test uptake in low- and middle-income countries. The quantitative data examines which intervention strategy is more or less effective to increase HIV test uptake, and the qualitative data can lend greater insights into the mechanisms and contextual factors involved [23]. We present the methods of this review in five steps, which are summarized below [24]:

### Step 1: formulating preliminary theoretical framework

Pawson and Tilley [24] stated that a realist approach first articulates the underlying theoretical framework about how the intervention strategies might work. To develop the theoretical framework, we conducted a scoping review of grey and published literature, including reports of the United Nations Program on HIV/acquired immunodeficiency syndrome (AIDS) (UNAIDS) and World Health Organization (WHO), previous reviews, and some theoretical and empirical research articles, that have specified about HIV stigma and HIV stigma reduction intervention strategies.

Our scoping review of the literature, at first, uncovered stigma reduction intervention strategies that have been implemented and tested in practice. We found that the intervention strategies that were used in the previous reviews, namely information-based approaches, coping skills acquisition, counseling approaches, contact with affected groups, structural approaches, and biomedical interventions, were mostly targeting the people living with HIV [6, 8]. As the outcome of interest of this review is HIV test uptake, we intend to focus on intervention strategies that target the general population, unaware of their HIV status. Scambler’s hidden distress model states that people with a stigmatizing condition initially develop “felt stigma”, in which they fear of potential discrimination, and so choose a strategy of non-disclosure and concealment [25]. An initial consequence of HIV stigma is the fear of disclosure, which is negatively associated with HIV test uptake among the people who are unaware of their HIV status [2, 3]. Based on Scambler’s hidden distress model, Weiss proposed developing stigma reduction intervention strategies that target both the people with a stigmatizing condition and the general population that are unaware of their HIV status [9]. Therefore, we used Weiss’s categories of intervention strategies (based on Scambler’s hidden distress model) to identify three types of intervention strategies that target a general population, namely

1. interventions to create awareness,
2. interventions to provide support; and
3. interventions to develop laws and normative behavior [9] (see Table 1).

**Table 1:** Stigma reduction intervention strategies

Intervention strategies	Definition	Interventions
Create awareness	Interventions having HIV-specific fact-based information-based written or verbal communication and education as a major component	Peer education, in-depth discussion, lecture, role play, interactions, advertisement, radio broadcast, school curriculum
Provide support	HIV-specific interventions that provide support to the people living with or associated with or at risk of HIV/AIDS in four domains: Psychosocial, clinical, socio-economic, and family and community	One-to-one counseling, empathy instruction, group counseling, support groups, training, access to treatment, nutritional support
Develop laws and normative behavior	Interventions that incorporate HIV-specific legislation that protects and respects the human rights of people living with HIV and supersedes negative customary laws and also the interventions related to increase community organizing and actions	Developing platforms to discuss stigma, providing compensation, community meeting, community organizing, laws, health policies

**Fig. 1:**
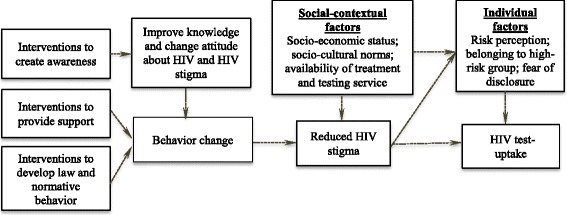
Preliminary theoretical framework

After identifying the intervention strategies, we developed a theoretical framework (see Fig. 1) that explains the potential mechanisms of how the intervention strategies influence HIV test uptake. In Fig. 1, the first three boxes are the intervention strategies and the dashed arrows that connect the boxes represent the potential mechanisms through which these individual intervention strategies might increase HIV test uptake. For example, the interventions that are designed to create awareness reduce stigma through increasing knowledge and changing attitudes about HIV. Likewise, the interventions to provide support and develop laws and normative behavior reduce stigma through changing the stigmatizing behavior. It can be assumed that a reduced level of stigma might increase HIV test uptake. However, this process might be influenced by different social-contextual and individual factors. For instance, increased knowledge on HIV might change misperceptions about HIV and HIV testing and, as such, reduce HIV stigma and increase HIV test uptake.

### Step 2: search strategy

To bring together different sources of evidence that supports, refines, or refutes our preliminary theoretical framework, we will perform an electronic database search in the following databases: PubMed, Excerpta Medica Database (EMBASE), POPLINE, PsycINFO, Sociological Abstracts, Web of Science, Scopus, and the Cumulative Index to Nursing and Allied Health Literature (CINAHL). Given that grey literature is a relevant source of information for realist reviews, we will also perform electronic searches in “Google scholar,” 3ie database, trail registers from Campbell International Development Coordinating Group, and databases of two international organizations, namely WHO and UNAIDS for program reports, evaluation reports, or policy documents. The search will be conducted using an iterative, purposive searching process. Snowball techniques will be used to identify additional studies from primary studies that might suggest other contextual influences and descriptors of mechanisms. To increase the comprehensiveness of our search strategy, a medical librarian will be consulted to improve our keyword search strategy. The search will stop when there is sufficient evidence to reasonably claim that the final theoretical framework is plausible (the draft keyword search strategy can be found in Additional file 1).

### Step 3: study selection criteria

Documents will be included in the review based on relevance, that is, the extent to which they inform development of the preliminary theoretical framework or clarify the CMO configurations [26]. We use five core inclusion criteria: (1) papers related to HIV-related interventions that should primarily address actionable causes of HIV stigma or have some components to reduce HIV stigma, (2) should be based in the low- and middle-income countries, (3) should be quantitative, qualitative, and mixed-method studies or program reports and policy documents that describe about various forms of stigma or describe about comparisons between CMO configurations between different stigma reduction intervention strategies, (4) should be written in English, and (5) the outcome should relate to HIV test uptake or non-quantitative studies should focus on the impact of stigma on HIV test uptake. There will be no exclusions based on the target population and study quality.

Screening of title, abstracts, and keywords of the documents identified in the initial search will be performed. Two reviewers will independently assess the relevance of the content in identified records for our synthesis, by comparing the abstract against the following three criteria: (1) Does the abstract refer to stigma reduction? (2) Does the abstract describe HIV test uptake as a dependent outcome? and (3) Does the abstract report methods to test hypotheses related to our proposed mechanisms? Abstracts will be coded as “Yes” if all three-inclusion criteria are satisfied, as “Unclear” if the abstract satisfies at least one criterion, and “No” if none of the criteria are met. If no abstract is available, the title of articles will be screened for eligibility, and potentially relevant studies will be coded as “Unclear.”

After the initial screening of abstracts, the full text of articles coded as “Yes” and “Unclear” will be retrieved and evaluated by two independent reviewers for a second time to ensure that one or more of our inclusion criteria are met. Disagreements about articles to be included and excluded will be resolved through group consensus. The reasons for exclusion will be recorded.

### Step 4: data extraction

Data will be extracted from the articles based on a data extraction tool (see Additional file 2) by the lead author and checked by a second member of the subgroup. As the contents of the preliminary theoretical framework are embedded in the data extraction form, this will provide a template to interrogate “what works, for whom, in what circumstances.” When extracting data, if an article does not include information relevant to a question in the form, the extractor will record “Not reported.” Direct quotation from the article is considered very informative and will be accompanied by the page number from which the quote is taken [27]. As the aim of the data extraction process is to evaluate the preliminary theoretical framework, the content from each group’s data extraction tables will be incorporated to form evidence tables. To test the usability and functionality of the data extraction form, the tool will be pretested on two purposefully selected articles [27].

Assessment of the quality of papers will be performed using a mixed methods appraisal tool [26, 28]. This tool allows an assessment of quantitative and qualitative data and provides one tool for appraising the quality of diverse study designs [28]. Generally, in a realist review, it is likely that only a fragment rather than the entire study will inform the theoretical framework. Therefore, the tool will not be applied to the whole study but only to those aspects that relate to our theoretical framework. The information about the study quality will complement the synthesis process by informing whether a particular inference drawn from a primary study is based on sufficient evidence to make a methodologically credible contribution to the theoretical framework [29]. A table will be developed that summarizes authors, objectives, study type, different methodological aspects, and study country for all the studies included.

### Step 5: data analysis and synthesis

The analysis and synthesis will be based on the principles of realist evaluation and will follow the following steps [24, 27]:

1. Organizing extracted data into evidence tables: The data extracted from each study using the data extraction tool will be summarized and organized in one or more evidence tables. The evidence tables will also include the link back to the source papers.
2. Theming by individual reviewers: Quantitative data will be analyzed by computing pooled hazard ratios or risk ratios as appropriate, with 95 % confidence intervals for each intervention strategy using the R-software package. It is likely that there will be a significant heterogeneity among the studies in terms of interventions and populations. If heterogeneity among the studies cannot be controlled, the quantitative data will be analyzed narratively. Qualitative data will be coded and arranged into themes by two independent reviewers in the NVivo software package. Line-by-line coding of the findings section of the selected studies will be performed. Themes will be developed from the initial codes based on reoccurring ideas that are similar in meaning, and relationships will be identified between the codes. Themes are patterns across data sets that are important to the description of a phenomenon. Identified themes will then be discussed between the reviewers, and contrary evidence will be sought [30].
3. Formulating chains of inference from the identified themes: We will then look for chains of inference (connections) across extracted data and themes. This will follow an iterative process, in which connections will be looked for across data/themes to build up a cumulative picture. The two reviewers will jointly formulate the connections, and this information will be shared and discussed in the group.
4. Linking the chains of inference and developing generative mechanisms: The chains of inference will be linked together to develop potential mechanisms, contexts, and outcome chains (generative mechanisms). These generative mechanisms will act as synthesized statements of findings and will be confirmed by returning to the source evidence. Patterns of similar mechanisms will then be compared across different contexts to see if similar outcomes are generated and the theoretical framework will be improved, if necessary, if new CMO configurations arise. All these interpretive processes will be performed through the discussion and agreement in the group.
5. Refining the preliminary theoretical framework: Finally, the preliminary theoretical framework will be refined to reflect the generative mechanisms that are supported by evidence. In the final theoretical framework, arrow thickness will be used to reflect the relative strength of the evidence, and dashed connecting lines will indicate hypothesized configurations of context, mechanism, and outcome [31].

### Knowledge dissemination

Results of this study will be disseminated to academic and non-academic audiences through peer-reviewed publications, conferences, and formal and informal presentations to policymakers and practitioners. Evidence generated from this synthesis will be used to inform the development of theory-driven, evidence-based interventions aimed at preventing HIV transmission through increasing HIV test uptake.

## Discussion

This realist review will be the first to unpack stigma reduction and HIV test uptake into CMO configurations. The realist approach will offer both a quantitative and a qualitative exploration of the available evidence to develop and propose a theoretical framework that explains why and how HIV stigma reduction intervention strategies influence HIV test uptake in low- and middle-income countries. The realist synthesis will also contribute to understanding the contextual factors that may mediate or moderate intervention outcomes, further informing the development of robust interventions [31].

In addition to offering a more exhaustive assessment of published quantitative and qualitative studies, and grey literature, this review will supersede existing reviews by including substantially more interventions. Moreover, the previous reviews [6, 8] included studies that were mostly conducted in high-income countries; the current review will specifically include studies from low- and middle-income countries to explain how the context determines the effect of stigma reduction intervention strategies on HIV test uptake. The focus on low- and middle-income country studies has been inspired by (1) the fact that HIV and HIV stigma mostly prevail in these countries and (2) the assumption that mechanisms related to social stigma differ for these countries as opposed to other countries. Insights from a context-specific approach may be less transferable to other countries, but they do provide more relevant information to local professionals [32]. Such knowledge has the great potential to guide policymakers on which contexts to modify or what kind of resources to enable, which in turn, activates the mechanisms that generate desired outcomes.

Due to an iterative and purposive search procedure, a limitation of the realist evaluation methodology might be that it is harder to reproduce as the selection and development of the theoretical framework is based on judgment [33]. Nevertheless, to minimize this limitation, we will follow a very transparent process developing a summary table including the methodological details and link back to the source papers. We will include papers written in English only, which may lead to information bias, although evidence that proofs this point is still scarce. As the findings from a realist review are theoretically transferable across one or more contexts, we believe that the results of this review might be imperative to design appropriate strategies for the success of overall AIDS response in low- and middle-income countries.

## Additional files

Additional file 1:
**Draft keyword search strategy.** Provides an overview of keyword serch strategy used in several databases in thisstudy. (DOCX 54 kb) Additional file 2:
**Summary of data extraction tool.** (DOCX 196 kb)

## Abbreviations

AIDS: acquired immunodeficiency syndrome
CMO: context-mechanism-outcome
HIV: human immunodeficiency virus
UNAIDS: United Nations Program on HIV/AIDS
WHO: World Health Organization
