# Supplementary material for: Effect of stigma reduction intervention strategies on HIV test uptake in low- and middle-income countries: a realist review protocol
Source: Syst Rev. 2015 Nov 2;4:142. doi: 10.1186/s13643-015-0130-3 (PMC4630912; doi:10.1186/s13643-015-0130-3)
Supplement: Additional file 1: — Draft keyword search strategy. Provides an overview of keyword serch strategy used in several databases in thisstudy. (DOCX 54 kb) [file 13643_2015_130_MOESM1_ESM.docx]

Additional file 1

**Draft keyword search strategy**

“HIV” OR “Human immuno-deficiency virus”, OR “Acquired immuno-deficiency Syndrome” OR “AIDS” OR “HIV/AIDS”

AND

“Stigma” OR “discrimination” OR “Blame” OR “Shame” OR “Attitude” OR “stereotype” OR “fear” OR “prejudice”

AND

“Interventions” OR “programs” OR “reducing stigma” OR “counseling” OR “pamphlet” OR “communication” OR “training” OR “peer education” OR “health education” OR “visual information” OR “group discussion” OR “media advertisement” OR “behavior change” OR “attitude change” OR “stigma reduction”

AND

“HIV VCT” OR “HIV testing” OR “voluntary counseling and testing” OR “HIV counseling and testing” OR “HCT” OR “HIV voluntary counseling and testing” OR “HIV anonymous counseling and testing” OR “HIV ACT”

Additional file 2

**Summary of data extraction tool**

1. Study identification details: Authors, title, location, publication date and date of study
2. Intervention characteristics and objectives: Details of what was done in a program, duration, target group, study type, comparison
3. Outcome and study objective: What is the outcome (stigma reduction/HIV test-uptake)?
4. Description of association between intervention and outcome; effect sizes (or risk ratios)
5. Contexts: For whom (target population)? In what circumstances? Where?
6. Contextual factors reported to be of influence: Individual and social-contextual factors
7. Mechanism or reported underlying assumptions: Descriptions about how and why the program may have worked in particular contexts
8. Mechanism is based on: Is the evidence of the mechanism based on: empirical evidence, reference to literature, or author opinion/speculation in the discussion
9. Mechanism Number: Does this mechanism in the article reflected in the mechanisms from the initial framework? If yes, assign mechanism with the associated number from the initial framework (e.g., M1, M2)
10. Page: Page number from which the mechanism is drawn
11. Paragraph: Paragraph on the page from which the findings/mechanism is drawn
12. Starts with‘‘…’’: First few words of sentence from which the mechanism is drawn
13. Richness: Available description of the mechanism
14. Evaluation: time of the evaluation, level of evaluation and method of evaluation
15. Additional articles: References found in the article that may lead to new mechanisms
16. Comments: General comments/ comments about the article
