# Supplementary material for: Effect of stigma reduction intervention strategies on HIV test uptake in low- and middle-income countries: a realist review protocol
Source: Syst Rev. 2015 Nov 2;4:142. doi: 10.1186/s13643-015-0130-3 (PMC4630912; doi:10.1186/s13643-015-0130-3)
Supplement: Additional file 2: — Summary of data extraction tool. (DOCX 196 kb) [file 13643_2015_130_MOESM2_ESM.docx]

Effect of stigma reduction intervention strategies on HIV test uptake: developing a preliminary theoretical framework

# Abstract

## Introduction: HIV stigma is a major barrier for HIV testing. It is, therefore, important that effective stigma reduction intervention strategies are identified and a conceptual framework explaining the mechanisms how the intervention strategies increase HIV testing rate be developed.

## Methods: To develop a preliminary conceptual framework that illustrates the mechanisms how stigma reduction intervention strategies increase HIV test uptake, we conducted a scoping review of grey and peer-reviewed literature. Unlike other traditional literature reviews, our review synthesized only the contents that informed the development of the theoretical framework or clarify the mechanisms identified. The data synthesis was performed in four steps: (1) mapping data sources to identify and select the literature; (2) extensive reading and rereading to develop the concepts; (3) organizing and categorizing the concepts; and (4) synthesizing concepts into a theoretical framework.

Results: The framework contains three intervention strategies: interventions to create awareness, to provide support, and to develop law and normative behavior. The interventions to create awareness might improve knowledge and change attitude about HIV and HIV stigma. This might reduce HIV stigma through changing stigmatizing behavior and as such, increase HIV test uptake. Likewise, the interventions to provide support and to develop laws and normative behavior might also reduce stigma through changing the stigmatizing behavior of the people and increase HIV test-uptake. However, the framework proposes that these mechanisms could be influenced by the interaction of various social-contextual and individual factors.

## Conclusion: This framework sheds new light on the study of effect of stigma reduction intervention strategies on HIV test uptake. We believe it will be potentially valuable for guiding a realist review.

## Key words: HIV-testing; Realist review; Scoping review; Stigma reduction intervention strategies; Theoretical framework.

# Introduction

The Joint United Nations Program on HIV/AIDS defined HIV stigma as a process of devaluation of people either living with or associated with HIV and AIDS [[1](#_ENREF_1)]. HIV/AIDS is likely to be stigmatized because it is generally perceived as dangerous, contagious and associated with behaviors that are outside the social norms [[2](#_ENREF_2)]. HIV stigma may lead to consequences, such as loss of friendship and family ties, dismissal from school and occupation, and denial from health care [[3](#_ENREF_3), [4](#_ENREF_4)]. Moreover, HIV stigma is associated with lower uptake of HIV testing services that leads to higher transmission rates [[5](#_ENREF_5), [6](#_ENREF_6)]. Therefore, it is important that intervention strategies that reduce HIV stigma and increase HIV test uptake be developed and implemented.

In recent years, there have been some progress in identifying the causes and dimensions of HIV stigma, and developing guidelines for the implementation and evaluation of stigma reduction interventions [[7](#_ENREF_7)]. However, development and implementation of these interventions have received much less attention. Also, these interventions are too seldom evaluated for effectiveness on uptake of HIV testing services [[7](#_ENREF_7)]. Moreover, systematic reviews synthesizing existing evidence on effect of stigma reduction interventions strategies on HIV test-uptake has not yet been documented.

Key gaps remain in the literature on the effectiveness of stigma reduction interventions on HIV test uptake. Thus, developing a conceptual framework or an evidence base that describes the mechanisms how interventions reduce HIV stigma and increase HIV test uptake is of paramount importance. A conceptual framework not only makes possible the better understanding of contextual factors that may mediate or moderate intervention outcomes, but also facilitates the development and implementation of robust interventions [[8](#_ENREF_8)]. Therefore, a realist synthesis of the existing evidence is needed to develop the framework that would clarify the mechanisms how stigma reduction intervention strategies impact on HIV test-uptake [[9](#_ENREF_9)].

Basically, a realist review starts from a literature review to develop a preliminary theoretical framework and then synthesizes existing qualitative and quantitative evidence to test and refine the framework [[9](#_ENREF_9)]. A scoping review of the literature is generally preferred when the topic has not yet been extensively reviewed or is of a complex or heterogeneous nature [[10](#_ENREF_10)]. Therefore, this scoping review was conducted as a preliminary step to a realist review to develop a preliminary theoretical framework illustrating potential mechanisms how stigma reduction intervention strategies increase HIV test uptake. Specifically, this scoping review was aimed at: (1) identifying different stigma reduction intervention strategies that have been implemented and tested in practice, and (2) developing a theoretical framework that illustrates the potential mechanisms that the intervention strategies follow to increase HIV test uptake.

# Methods

We conducted a scoping review of the literature to develop a theoretical framework that explains potential mechanisms how stigma reduction intervention strategies increase HIV test uptake. Grey and peer-reviewed literature were identified and purposefully selected through free text searching for the key words, such as “HIV”; “Stigma”; “Stigma reduction interventions” and “HIV testing”, on PubMed, Google Scholar and official websites of the Joint United Nations Program on HIV/AIDS and the World Health Organization. There was no restriction on study type.

We included papers related to: (a) HIV-related interventions that primarily addressed actionable causes of HIV stigma or have some components to reduce HIV stigma; (b) theoretical papers or empirical research articles or program reports or policy documents that described about various forms of stigma or different stigma reduction intervention strategies; and (c) the outcome related to HIV test uptake or impact of stigma on HIV test uptake.

Unlike other traditional literature reviews, we only synthesized the contents that informed the development of the theoretical framework or clarify the mechanisms identified in this review [[11](#_ENREF_11)]. It is likely that only a fragment rather than the entire study would inform the theoretical framework. A scoping review is generally considered to provide a descriptive overview of the reviewed material without critically appraising individual studies or synthesizing evidence and therefore, we only described the data that help develop the theoretical framework or clarify the mechanisms [[10](#_ENREF_10)]. For data synthesis, we did not follow any particular methods, such as thematic analysis, because of the nature of the review and short time frame available to us.

The following steps were followed for data synthesis [[12](#_ENREF_12)]: (1) mapping the selected data sources to identify and select the literature; (2) extensive reading and rereading to develop the concepts based on the similarity in meaning and based on our interpretation; (3) organizing and categorizing the concepts to rule out contradictory concepts and to integrate the similar concepts; and (4) synthesizing concepts into a theoretical framework though an iterative and open process. This whole process was repeated unless a general consensus on the final conceptual framework was reached after the discussion among the review team members.

# Results

### Existing stigma reduction intervention strategies

### *Brown’s categories of the intervention strategies*

Brown et al conducted a systematic review to examine the effectiveness of the interventions to reduce stigma in 2003. In this review, 21 different interventions were grouped into four strategies, namely information-based, coping skills, counseling, and contact with affected groups [[13](#_ENREF_13)]. Brown et al grouped all the interventions related to advertisement, information packs, or presentation in a class or lecture as information-based strategy [[13](#_ENREF_13)]. Likewise, the interventions that provided individuals who had a contact with people living with HIV/AIDS with relaxation and stress management skills through role play, group desensitization and master imagery were grouped as coping skills strategy [[13](#_ENREF_13)]. Counseling strategy included interventions, such as one to one counseling or group counseling, in which HIV-related information was provided and participants were allowed to have intimate discussions [[13](#_ENREF_13)]. Contact strategy included interventions that provided an opportunity for people to interact with and to visualize being people living with HIV/AIDS.

Brown’s categories of intervention strategies was followed by Sengupta et al in 2011 [[14](#_ENREF_14)] and Stangl et al in 2013 [[15](#_ENREF_15)]. Stangle et al [[15](#_ENREF_15)] added two more strategies, namely biomedical and socio-structural interventions. Structural interventions included interventions that aimed at removing, reducing or altering for the better structural factors that influence the stigmatization process, such as laws that criminalize HIV, hospital or workplace policies that institutionalize discrimination of people living with HIV/AIDS [[15](#_ENREF_15)]. Where as, biomedical interventions included the interventions related to health services utlization, such as availability of antiretroviral therapy, availability of testing and counseling service [[15](#_ENREF_15)].

## Brown et al suggested that most of the stigma reduction interventions aimed at increasing tolerance of the people living with HIV/AIDS among segment of general population, increasing willingness to treat people living with HIV/AIDS among health care providers and improving coping strategies among people living with HIV/AIDS [[13](#_ENREF_13)]. The other evaluation studies also included the interventions that targeted mostly the people living with or associated with HIV and AIDS [[14](#_ENREF_14), [15](#_ENREF_15)]. Thus, to understand the impact of stigma reduction intervention strategies on HIV test uptake among the general population, Brown’s categories might be inadequate.

### *Scambler’s hidden distress model*

Scambler’s hidden distress model is based on the results of a study that aimed at understanding the experiences and coping strategies of people with epilepsy. This model has summarized three propositions that are based on the distinction between the two types of stigma, namely felt stigma and enacted stigma [[16](#_ENREF_16), [17](#_ENREF_17)]. First, due to a stigmatizing condition, people develop a felt stigma, in which they fear of potential discrimination. Second, due to the fear of potential discrimination, they choose a strategy of non-disclosure and concealment. And last, the net effect of this strategy is that felt stigma is typically more disruptive than enacted stigma.

Based on the first and second propositions of the model, the negative association between HIV stigma and HIV test uptake can be understood by the fact that an initial consequence of felt stigma is the adoption of non-disclosure and concealment of the condition. For example, due to the fear of stigma of a positive test result, people choose the strategy to non-disclosure and concealment, and keep avoiding the test. Likewise, based on the third proposition of the model, avoiding an HIV test could prove more disruptive, as it increases the risk of transmission.

Scambler’s hidden distress model suggests that stigma is produced due to perceived differences in individual experiences, social attributes and power between stigmatized and perpetrator [[17](#_ENREF_17)]. Thus, the intervention strategies should include the interventions that target both general population and people living with HIV/AIDS [[18](#_ENREF_18), [16](#_ENREF_16)]. Weiss based on Scambler hidden distress model to develop three different stigma reduction intervention strategies: interventions to create awareness; interventions to provide support; and interventions to develop laws and normative behavior [[18](#_ENREF_18), [16](#_ENREF_16)]. To develop the framework, we based on these three strategies to group stigma reduction interventions [[16](#_ENREF_16)] (See Table 1).

### Theoretical framework for effect of stigma reduction intervention strategies to increase HIV-test uptake

We developed a theoretical framework that explains the potential mechanisms how the intervention strategies influence HIV test-uptake (see Figure 1). The first three boxes shown in the figure are the intervention strategies, namely interventions to create awareness, interventions to provide support, and interventions to develop law and normative behavior. We defined interventions to create awareness as the interventions having HIV-specific fact-based information based written or verbal communication and education as a major component [[13](#_ENREF_13)]. These interventions included peer education, in-depth discussion, lecture, role-play, interactions, radio broadcast, advertisement and school curriculum.

Similarly, the interventions to provide support was defined as the HIV-specific interventions that provide support to the people living with or associated with or at risk of HIV and AIDS on four domains, namely psychosocial; clinical; socio-economic; and family and community [[19](#_ENREF_19)]. These interventions included one-to-one counseling, empathy instruction, group counseling, support groups, training, access to treatment and nutritional support. The interventions to develop laws and normative behavior was the intervention strategy that deals with HIV-specific legislations and policies that protect and respect the human rights of people living with HIV and supersed negative customary laws and also the interventions-related to increase community organizing and actions [[19](#_ENREF_19)]. These interventions included developing platforms to discuss stigma, providing compensation, community meeting, community organizing, laws and health policies.

In the framework, the dashed arrows connect the boxes to represent the potential mechanisms that these individual intervention strategies follow through to increase HIV test-uptake. There are two possible mechanisms identified. First, the interventions that are designed to create awareness might improve knowledge and change attitude about HIV and HIV stigma. This might reduce HIV stigma through changing stigmatizing behavior and as such increase HIV test uptake. Next, the interventions to provide support and to develop laws and normative behavior might also reduce stigma through changing the stigmatizing behavior of the people. The reduced level of stigma might increase HIV test-uptake.

According to the framework, it is likely that the effect of stigma reduction interventions on HIV test-uptake behavior of people might be influenced by various social-contextual factors [[20-23](#_ENREF_20)]. For example, social-contextual factors, such as poverty, illiteracy, lack of availability of treatment, cultural and gender norms might influence the intervention process and the association between stigma reduction and HIV test uptake [[20-23](#_ENREF_20)]. Moreover, the framework illustrates that the social-contextual factors, such as socio-economic status and socio-cultural norms, might influence individual factors, such as risk behaviors, risk perception and fear of disclosure, and thus, the interaction between both may also influence the effect of stigma reduction intervention strategies on HIV test uptake [[20-23](#_ENREF_20)].

# Discussion

Our theoretical framework sheds new light on the study of effect of stigma reduction intervention strategies on HIV test uptake. To our knowledge, this is one of the first theoretical frameworks that specifically illustrate the potential mechanisms that the stigma reduction intervention strategies would follow to increase HIV test-uptake.

To develop the framework, we did not refer to Brown’s categories of the intervention strategies because most of the interventions included were targeting people living with or associated with HIV and AIDS only [[13-15](#_ENREF_13)]. Besides, we felt the need of a categorization that would include the interventions targeting both the people living with or associated with HIV and AIDS and the general population who do not know about their HIV status. Therefore, we based on Scambler's hidden distress model that was later extended by Weiss to group different stigma reduction interventions into three strategies: interventions to create awareness, interventions to provide support, and interventions to develop law and normative behavior [[18](#_ENREF_18)].

Link and Phelan stated that interventions should focus on more than one strategies at a time to reduce stigma [[24](#_ENREF_24)]. In the previous studies, almost all of the stigma reduction interventions that have been tested have also incorporated more than one strategies and the most common to all was awareness-raising strategy [[13-15](#_ENREF_13)]. However, it has not been clear whether the awareness raising or the other intervention strategies is more effective to reduce stigma and generate outcomes. Besides, most interventions that were included in the previous studies stressed on mitigating enacted stigma [[13-15](#_ENREF_13)]. But if Scambler’s model applies, interventions that stress on felt stigma should also be tested for effectiveness [[16](#_ENREF_16)]. We believe that this framework will be potentially valuable for guiding a realist review that would include wide range of interventions to uncover which intervention strategies are more or less effective to reduce stigma and increase HIV test uptake.

Studies [[13](#_ENREF_13), [25](#_ENREF_25), [26](#_ENREF_26)] from high-income countries have reported that the stigma reduction intervention strategies have not only been effective to reduce stigma, but also to increase HIV test-uptake. Where as, studies conducted in low and middle-income countries indicate that such intervention strategies are effective to increase HIV disclosure and safer sex practices, but not to increase HIV test-uptake [[27-29](#_ENREF_27)]. It might be that the social-contextual factors, such as culture, resources, opportunities and levels of sophistication largely vary between high-income and low-income economies and this can largely influence the implementation process and effectiveness of the interventions [[17](#_ENREF_17)]. In consistent with Link and Phelan’s model [[24](#_ENREF_24)], our framework also suggested that effectiveness of the interventions are likely to be mediated or moderated by various social-contextual factors.

So far, most of the stigma reduction interventions are developed and tested in high-income countries [[13](#_ENREF_13)]. Information about effectiveness of stigma reduction intervention strategies that are developed and tested in the low and middle income countries has not been understood [[30](#_ENREF_30)]. The low and middle income country studies should be focused due to: [1] the fact that HIV and HIV stigma mostly prevail in these countries and [2] the assumption that mechanisms related to social stigma differ for these countries as opposed to other countries. Insights from a context-specific approach may be less transferable to other countries, but they do provide more relevant information to local professionals [[31](#_ENREF_31)]. Such knowledge has the great potential to guide policymakers on which contexts to modify or what kind of resources to enable, which in turn, activates the mechanisms that generate desired outcomes.

### Limitations

Due to purposive search procedure, a limitation of this scoping review might be that it is harder to reproduce as the selection of the articles and development of the framework is based on judgment [[32](#_ENREF_32)]. It was unrealistic to retrieve and screen all the relevant literature in this review due to the nature of the review and limited time available to us. Whatsoever, this is a possibility that this review may have missed some important studies. The lack of critical appraisal of included studies might influence our study results and therefore, should be cautiously used for program purpose. However, we believe that this framework will be potentially valuable to guide a realist review.

### Conclusions

This scoping review was conducted as a preliminary step to a realist review to develop a preliminary theoretical framework illustrating potential mechanisms how stigma reduction intervention strategies increase HIV test uptake. The framework suggested that the interventions that are designed to create awareness might improve knowledge and change attitude about HIV and HIV stigma, and this might reduce HIV stigma through behavior change and as such, increase HIV test uptake. Likewise, the interventions to provide support and to develop laws and normative behavior might also reduce stigma through changing the stigmatizing behavior of the people and increase HIV test-uptake. It is likely that these mechanisms might be mediated or moderated by the interaction of various social-contextual and individual factors. This theoretical framework, after being tested and refined by the realist review, can guide program managers to identify and implement the most effective stigma reduction intervention strategies to increase HIV test-uptake and consequently, to reduce the rate of HIV transmission, especially in low and middle income countries.

# References:

1. Unaids. UNAIDS fact sheet on stigma and discrimination2003.

2. Herek GM, Mitnick L, Burris S, Chesney M, Devine P, Fullilove MT et al. Workshop report: AIDS and stigma: a conceptual framework and research agenda. AIDS & public policy journal. 1998;13(1):36-47.

3. Kalichman SC, Simbayi LC. HIV testing attitudes, AIDS stigma, and voluntary HIV counselling and testing in a black township in Cape Town, South Africa. Sexually transmitted infections. 2003;79:442-7. doi:10.1136/sti.79.6.442.

4. Young SD, Nussbaum AD, Monin BÆ. Potential moral stigma and reactions to sexually transmitted diseases: evidence for a disjunction fallacy. Personality and social psychology bulletin. 2007;33:789-99. doi:10.1177/0146167207301027.

5. Golub SA, Gamarel KE. The impact of anticipated HIV stigma on delays in HIV testing behaviors: findings from a community-based sample of men who have sex with men and transgender women in New York City. AIDS patient care and STDs. 2013;27(11):621-7. doi:10.1089/apc.2013.0245.

6. Rintamaki LS, Davis TC, Skripkauskas S, Bennett CL, Wolf MS. Social stigma concerns and HIV medication adherence. AIDS patient care and STDs. 2006;20(5):359-68. doi:10.1089/apc.2006.20.359.

7. Unaids. Key programmes to reduce stigma and discrimination and increase access to justice in national HIV responses2012.

8. Brown HE, Atkin AJ, Panter J, Corder K, Wong G, Chinapaw MJM et al. Family-based interventions to increase physical activity in children: a meta-analysis and realist synthesis protocol. BMJ open. 2014;4(8):e005439-e. doi:10.1136/bmjopen-2014-005439.

9. Pawson R, Tilley N. Realistic Evaluation. London: Sage publication; 1997.

10. Pham MT, Rajić A, Greig JD, Sargeant JM, Papadopoulos A, McEwen SA. A scoping review of scoping reviews: advancing the approach and enhancing the consistency. Research synthesis methods. 2014;5(4):371-85. doi:10.1002/jrsm.1123.

11. Durham J, Bains A. Research protocol: a realist synthesis of contestability in community-based mental health markets. Systematic reviews. 2015;4(1):32-. doi:10.1186/s13643-015-0025-3.

12. Jabareen Y. Building a conceptual frmaework: philosophy, definitions, and procedure International Journal of Qualitative Methods. 2009;8(4):49-62.

13. Brown L, Macintyre K, Trujillo L. Interventions to reduce HIV/AIDS stigma: What have we learned? 2003. p. 49-69.

14. Sengupta S, Banks B, Jonas D, Miles MS, Smith GC. HIV interventions to reduce HIV/AIDS stigma: A systematic review. 2011. p. 1075-87.

15. Stangl AL, Lloyd JK, Brady LM, Holland CE, Baral S. A systematic review of interventions to reduce HIV-related stigma and discrimination from 2002 to 2013: how far have we come? 2013.

16. Cross HA, Heijnders M, Dalal A, Sermrittirong S, Mak S. Interventions for Stigma Reduction ‚Äì Part 1: Theoretical Considerations. Disability, CBR &amp; Inclusive Development; Vol 22, No 3 (2011): Winter. 2012.

17. Scambler G Fau - Heijnders M, Heijnders M Fau - van Brakel WH, van Brakel WH. Understanding and tackling health-related stigma. PsycholHealth Med. 2006(1354-8506 (Print)).

18. Weiss MG, editor. Stigma and the social burden of neglected tropical diseases2008 2008.

19. Group CaSW. What do we really mean by HIV care and support: progress toward comprehensive definition. London2008.

20. Castro A, Farmer P. Understanding and addressing AIDS-related stigma: From anthropological theory to clinical practice in Haiti. 2005. p. 53-9.

21. Florom-Smith AL, De Santis JP. Exploring the concept of HIV-related stigma. Nursing forum.47(3):153-65. doi:10.1111/j.1744-6198.2011.00235.x.

22. Gerbert B, Maguire BT, Bleecker T, Coates TJ, McPhee SJ. Primary care physicians and AIDS. Attitudinal and structural barriers to care. JAMA : the journal of the American Medical Association. 1991;266:2837-42. doi:10.1001/jama.266.20.2837.

23. Hamra M, Ross MW, Karuri K, Orrs M, D'Agostino A. The relationship between expressed HIV/AIDS-related stigma and beliefs and knowledge about care and support of people living with AIDS in families caring for HIV-infected children in Kenya. AIDS care. 2005;17(7):911-22. doi:10.1080/09540120500100593.

24. Link BG, Phelan JC. Conceptualizing Stigma. Annual Review of Sociology. 2001;27(1):363-85. doi:10.1146/annurev.soc.27.1.363.

25. Perry S, Fishman B, Jacobsberg L, Young J, Frances A. Effectiveness of psychoeducational interventions in reducing emotional distress after human immunodeficiency virus antibody testing1991.

26. Simpson WM, Johnstone FD, Boyd FM, Goldberg DJ, Hart GJ, Prescott RJ. Uptake and acceptability of antenatal HIV testing: randomised controlled trial of different methods of offering the test1998. Report No.: 0959-8138 (Print)\n0959-535X (Linking).

27. Kaleeba N, Kalibala S, Kaseje M, Ssebbanja P, Anderson S, van Praag E et al. Participatory evaluation of counselling, medical and social services of The AIDS Support Organization (TASO) in Uganda. AIDS care. 1997;9(1):13-26. doi:10.1080/09540129750125307.

28. Kerry K, Margie C. Cost effective AIDS awareness programme on commercial farms in Zimbabwe. IntConfAIDS. 1996;11:45-.

29. Kiguli AR. Community initiatives against HIV, care for PWA and care for AIDS affected ones: a multi- cultural response. IntConfAIDS. 1996;11:472-.

30. Keusch GT, Wilentz J, Kleinman A. Stigma and global health: developing a research agenda. Lancet. 2006;367(9509):525-7. doi:10.1016/s0140-6736(06)68183-x.

31. Hannes K, Harden A. Multi-context versus context-specific qualitative evidence syntheses: combining the best of both. Research synthesis methods. 2011;2(4):271-8. doi:10.1002/jrsm.55.

32. Wong G, Greenhalgh T, Westhorp G, Buckingham J, Pawson R. RAMESES publication standards: Meta-narrative reviews. Journal of Advanced Nursing. 2013;69(5):987-1004. doi:10.1111/jan.12092.
